# Monitoring of Tumor Growth with [^18^F]-FET PET in a Mouse Model of Glioblastoma: SUV Measurements and Volumetric Approaches

**DOI:** 10.3389/fnins.2016.00260

**Published:** 2016-06-14

**Authors:** Adrien Holzgreve, Matthias Brendel, Song Gu, Janette Carlsen, Erik Mille, Guido Böning, Giorgia Mastrella, Marcus Unterrainer, Franz J. Gildehaus, Axel Rominger, Peter Bartenstein, Roland E. Kälin, Rainer Glass, Nathalie L. Albert

**Affiliations:** ^1^Department of Nuclear Medicine, University Hospital of Munich, Ludwig Maximilians University of MunichMunich, Germany; ^2^Department of Neurosurgery, University Hospital of Munich, Ludwig Maximilians University of MunichMunich, Germany

**Keywords:** [^18^F]-FET PET, partial volume effect correction (PVEC), biological tumor volume (BTV), tumor growth monitoring, orthotopic glioblastoma mouse model (GL261)

## Abstract

Noninvasive tumor growth monitoring is of particular interest for the evaluation of experimental glioma therapies. This study investigates the potential of positron emission tomography (PET) using *O*-(2-^18^F-fluoroethyl)-L-tyrosine ([^18^F]-FET) to determine tumor growth in a murine glioblastoma (GBM) model—including estimation of the biological tumor volume (BTV), which has hitherto not been investigated in the pre-clinical context. Fifteen GBM-bearing mice (GL261) and six control mice (shams) were investigated during 5 weeks by PET followed by autoradiographic and histological assessments. [^18^F]-FET PET was quantitated by calculation of maximum and mean standardized uptake values within a universal volume-of-interest (VOI) corrected for healthy background (SUVmax/BG, SUVmean/BG). A partial volume effect correction (PVEC) was applied in comparison to *ex vivo* autoradiography. BTVs obtained by predefined thresholds for VOI definition (SUV/BG: ≥1.4; ≥1.6; ≥1.8; ≥2.0) were compared to the histologically assessed tumor volume (*n* = 8). Finally, individual “optimal” thresholds for BTV definition best reflecting the histology were determined. In GBM mice SUVmax/BG and SUVmean/BG clearly increased with time, however at high inter-animal variability. No relevant [^18^F]-FET uptake was observed in shams. PVEC recovered signal loss of SUVmean/BG assessment in relation to autoradiography. BTV as estimated by predefined thresholds strongly differed from the histology volume. Strikingly, the individual “optimal” thresholds for BTV assessment correlated highly with SUVmax/BG (ρ = 0.97, *p* < 0.001), allowing SUVmax/BG-based calculation of individual thresholds. The method was verified by a subsequent validation study (*n* = 15, ρ = 0.88, *p* < 0.01) leading to extensively higher agreement of BTV estimations when compared to histology in contrast to predefined thresholds. [^18^F]-FET PET with standard SUV measurements is feasible for glioma imaging in the GBM mouse model. PVEC is beneficial to improve accuracy of [^18^F]-FET PET SUV quantification. Although SUVmax/BG and SUVmean/BG increase during the disease course, these parameters do not correlate with the respective tumor size. For the first time, we propose a histology-verified method allowing appropriate *individual* BTV estimation for volumetric *in vivo* monitoring of tumor growth with [^18^F]-FET PET and show that standardized thresholds from routine clinical practice seem to be inappropriate for BTV estimation in the GBM mouse model.

## Introduction

The last decade has seen a considerable range of studies emphasizing the increasing importance of positron emission tomography (PET) using the radiolabeled amino acid analog *O*-(2-[^18^F]-fluoroethyl)-L-tyrosine ([^18^F]-FET) for clinical neuro-oncology (La Fougere et al., 2011; Dunet et al., 2012; Galldiks et al., 2015b). With precision medicine in mind, [^18^F]-FET PET has become of crucial interest especially for individual therapy management in glioma patients, since it visualizes the metabolic activity of the glioma-affected brain tissue and thereby broadens neuroimaging to the molecular level (Pauleit et al., 2005; Rachinger et al., 2005; Langen et al., 2006; Dhermain et al., 2010; Galldiks et al., 2012a). Whereas conventional imaging techniques like structural magnetic resonance imaging (MRI)—as the current clinical standard for neuro-oncological imaging—focus on morphologic information, [^18^F]-FET PET is a tool to visualize the altered biology of the tumoral lesion (as compared to non-neoplastic parenchyma) and promotes a personalized therapy management. In the clinical setting, [^18^F]-FET PET has proven its usefulness at primary diagnosis, e.g. for differential diagnosis (Hutterer et al., 2013; Rapp et al., 2013), tumor grading (Popperl et al., 2007; Jansen et al., 2012a; Lohmann et al., 2015), biopsy and treatment planning (Stockhammer et al., 2009; Plotkin et al., 2010; Kunz et al., 2011; Tonn et al., 2012), as well as for disease monitoring, e.g. for response assessment after different therapies (Piroth et al., 2011; Galldiks et al., 2012b, 2013b), early detection of tumor recurrence and differentiation from post-therapeutic changes (Mehrkens et al., 2008; Galldiks et al., 2015a).

While [^18^F]-FET PET therefore has the potential to play a more and more important role for neuro-oncology in the clinical setting, its pre-clinical potential is not yet fully appreciated. Pre-clinical brain tumor imaging faces similar difficulties as its clinical equivalent concerning e.g. reliable *in vivo* volumetry or bias due to post-therapy effects. Especially damage of the blood brain barrier (BBB) is a confounding factor for the evaluation of therapy effects for some of the most promising experimental therapeutics. Above all, pre-clinical glioma imaging in mouse models is challenging due to relatively small tumor sizes (of barely a few cubic millimeters), which require special diligence to precision imaging and encourage technically sophisticated methods of image correction.

Altogether, there is a need to improve pre-clinical *in vivo* imaging and tumor growth monitoring, when evaluating new experimental glioma therapies.

Despite the wide clinical use of [^18^F]-FET PET and in spite of its above suggested tremendous potential for pre-clinical research, only few animal studies—generally consisting in tracer comparisons or focusing on the chemical characteristics of [^18^F]-FET—have so far evaluated the performance of [^18^F]-FET imaging in rodent glioma models. To the author's knowledge, only two pre-clinical reports have so far described the use of longitudinal [^18^F]-FET PET for the purpose of brain tumor growth monitoring—both in the context of relative treatment response evaluation in xenograft glioblastoma (GBM) mouse models (Nedergaard et al., 2014, 2015). Studies focusing however on important methodological aspects of [^18^F]-FET PET, e.g. estimation of the biological tumor volume (BTV), are still lacking.

In this study, on the one hand, we evaluate the general feasibility of [^18^F]-FET PET in a murine GBM mouse model (GL261) using a volume of interest (VOI)-based approach for image analysis. We apply a partial volume effect correction (PVEC) to meet the small scale and finally improve accuracy of standard PET parameters. On the other hand, we aim to investigate the potential of [^18^F]-FET PET to reliably determine the BTV, which hasn't been investigated so far in the pre-clinical context. For this purpose, we first translated to our orthotopic GBM mouse model the clinical approach for BTV estimation in glioma patients consisting in a semiautomatic threshold-based VOI-calculation. Second, we evaluated this estimation by direct comparison of BTV to histologically determined tumor volume—an approach exclusively subject to pre-clinical investigations since it is not possible to reliably determine the histological tumor volume in glioma patients. Finally, we propose a method for BTV estimation using *individual* thresholds.

## Materials and methods

### Cell culture

GL261 as previously used (Markovic et al., 2009) were cultured in DMEM containing MEM non-essential amino acids (1x), 1:100 Penicillin-Streptomycin solution (all Life Technologies) and 10% FCS (Biochrome) and medium was changed every 2 days. Cell cultures were maintained in the incubator at 37°C in humidified and 5% CO_2_-conditioned atmosphere; cells were passaged when the cell density in the flask reached 80% confluence.

### Animal model

All experiments were performed in compliance with the National Guidelines for Animal Protection, Germany, with approval of the local animal care committee of the Government of Oberbayern (Regierung von Oberbayern), and overseen by a veterinarian.

Eight week old C57BL/6 mice were obtained from Charles River (Sulzfeld, Germany) and acclimated for 1 week. A first set of animal experiments was performed to analyze standard PET parameters and to determine optimal parameters for BTV measurements: At day 0, the mice were inoculated either with 50,000 GL261 cells suspended in 1 μL of saline (GBM mice, *n* = 15) or with 1 μL of saline for control (sham mice, *n* = 6). For inoculation, mice were anesthetized with i. p. injections of approximately 100 mg/kg ketamine 10% and 10 mg/kg xylazine 2% in 0.9% NaCl. Anesthetized mice were immobilized and mounted onto a stereotactic head holder (Kopf Instruments) in the flat-skull position. After surface disinfection with 7.5% Braunol solution (Braun) the skin of the skull was dissected with a scalpel blade. One millimeter anterior and 1.5 mm right to the bregma, the skull was carefully drilled with a 23-gauge needle tip. By stereotactic injection, 5 × 10^4^ cells or 1 μL PBS only applied with a 22-gauge Hamilton syringe (Hamilton Bonaduz,) 5 mm below the drill hole in the calvarium. Cells were slowly injected within 2 min and after a settling period of another minute the needle was removed in 1 mm steps per minute. After that, the wound was closed by suturing. Postoperative analgesia consisted of 100 μL of a 0.2% (w/v)-meloxicam solution i. p. while the mice were kept warm.

A second set of animal experiments was used to test the optimized parameters for BTV estimation: *N* = 15 supplementary GBM-bearing mice received PET scans and were all sacrificed for autoradiography and histology immediately after PET (*n* = 3 in week-3 and *n* = 4 in each week-2, -4, and -5).

### Study design

The GBM-bearing mice in the first set of animal experiments received weekly PET scans beginning with week-2 after inoculation. In addition, 3 shams were scanned in week-2 and 1 sham in each week-3, week-4, and week-5, irrespective of the longitudinal setup for GBM-bearing mice. Every week, three of the GBM-bearing mice and one corresponding sham were sacrificed for autoradiography and histology immediately after receiving the PET scan. The study was planned to end in week-6 with the last 3 GBM-bearing mice being sacrificed. A study overview is given in Table 1.

**Table 1:** **Modality overview**.

	**[^18^F]-FET PET schedule**		**[^18^F]-FET PET performed**		**Histology/AR performed**	
	**GBM**	**sham**	**GBM**	**sham**	**GBM**	**sham**
Week-2	15	3	14	3	3 (0)	1
Week-3	12	1	11	1	2 (2)	1
Week-4	9	1	6	1	3 (3)	1
Week-5	6	1	6	1	4 (3)	1
Week-6	3	1	0	0	0	0

*The middle row lists the effectively generated PET data per week as opposed to the previously intended PET schedule defined by the study design (shown in left row). The amount of effectively generated histologies/autoradiographies for comparison with PET data is specified in the right row. Number of GBM mice with confirmed tumor in histology is bracketed. AR = autoradiography*.

### PET

Mice received bolus injection of 14.7 ± 2.3 MBq of [^18^F]-FET in 150 μL of saline into the tail vein. [^18^F]-FET was used from clinical routine production (PET NET, Erlangen, Germany) as previously described (Wester et al., 1999). If not specified further, anesthesia was performed with isoflurane 1.5% delivered via a mask at 3.5 L/min in oxygen. Following placement in the tomograph (Siemens Inveon DPET), emission recording for the interval 0–40 min post injection (p. i.) followed by a 7 min transmission scan was obtained using a rotating [^57^Co] point source. The image reconstruction procedure consisted of three-dimensional ordered subset expectation maximization (OSEM) with four iterations and 12 subsets followed by a maximum *a posteriori* (MAP) algorithm with 32 iterations. Scatter and attenuation correction were performed and a decay correction for [^18^F] was applied. Frame setting consisted of two 20 min frames from which the latter (20–40 min post injection) was used for further analyses. The final voxel dimension of 0.39 × 0.39 × 0.80 mm was obtained by using a zoom factor of 1.0 and a 256 × 256 × 159 matrix. Manual rigid-body co-registration of PET images to a 3T magnetic resonance imaging (MRI) template, followed by manual rigid-body re-alignment of intra-individual longitudinal images, was accomplished using the PMOD fusion tool (version 3.4; PMOD Technologies Ltd.; Rominger et al., 2013).

**Figure 1:**
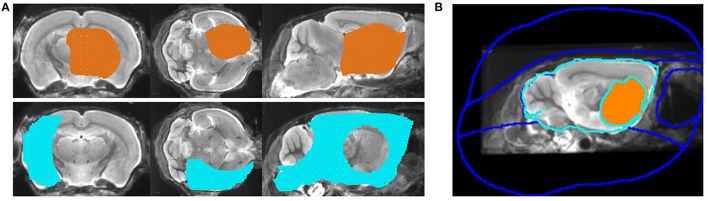
**(A)** A three-dimensional impression of both the universal tumor VOI surrounding the stereotactic coordinates (V = 88 mm^3^, upper row) and the background VOI placed in the tumor-free contralateral hemisphere (V = 123 mm^3^, lower row) is given. **(B)** Eight region VOI-mask used for PVEC. The “optimal” individual tumor VOI of this representative mouse (resulting from BTV analyses) is colored.

For VOI-based analysis, a universal VOI of 123 mm^3^ placed in the tumor-free contralateral hemisphere was set as background (BG) and a target VOI of 88 mm^3^ surrounding the stereotactic coordinates of the tumor in the right hemisphere served as universal tumor VOI (Figure 1A).

In a second approach, individual tumor VOIs were automatically drawn threshold-based and compared to the individual histology volume.

Image analysis comprised assessment of the mean standardized uptake value (SUVmean) and the maximal standardized uptake value (SUVmax) for both the universal and individual tumor VOI, each corrected for mean background activity (SUVmean/BG; SUVmax/BG).

### Autoradiography and histology

*Ex vivo* autoradiography was performed immediately after acquisition of PET data. Mice received intracardiac perfusion with PBS to wash out the blood followed by intracardiac perfusion with 4% PFA to fix the brain tissue (death within 65 min p. i.). The dissected brains, placed in a base mold and completely surrounded by cryo-matrix, were cooled down for < 5 min in a −80°C refrigerator, then frozen for approximately 10 more min at −20°C in a Leica CM 1510-1 Cryostat (Leica Microsystems, Nussloch, Germany) and cut in 16 μm thick horizontal sections. Every 24th section was mounted on glass slides within 75 min *post mortem*. An imaging plate (Fujifilm; BAS cassette2 2025) was exposed to the slides for 15 h, scanned at 25 μm resolution with Raytest equipment (CR 35 BIO, Dürr Medical, Germany), and analyzed with AIDA image analyzing software (V4.50). Regions of interest (ROIs) were manually drawn around the tumor and in the tumor-free contralateral hemisphere, and mean radioactivity concentrations per mg tissue equivalent were used for calculation of autoradiographic SUVmean/BG (mean of >15 slices per animal). Error-(%) in uncorrected and PVE-corrected *in vivo* PET results was calculated relative to this high resolution reference of autoradiographic results *ex vivo*.

**Figure 2:**
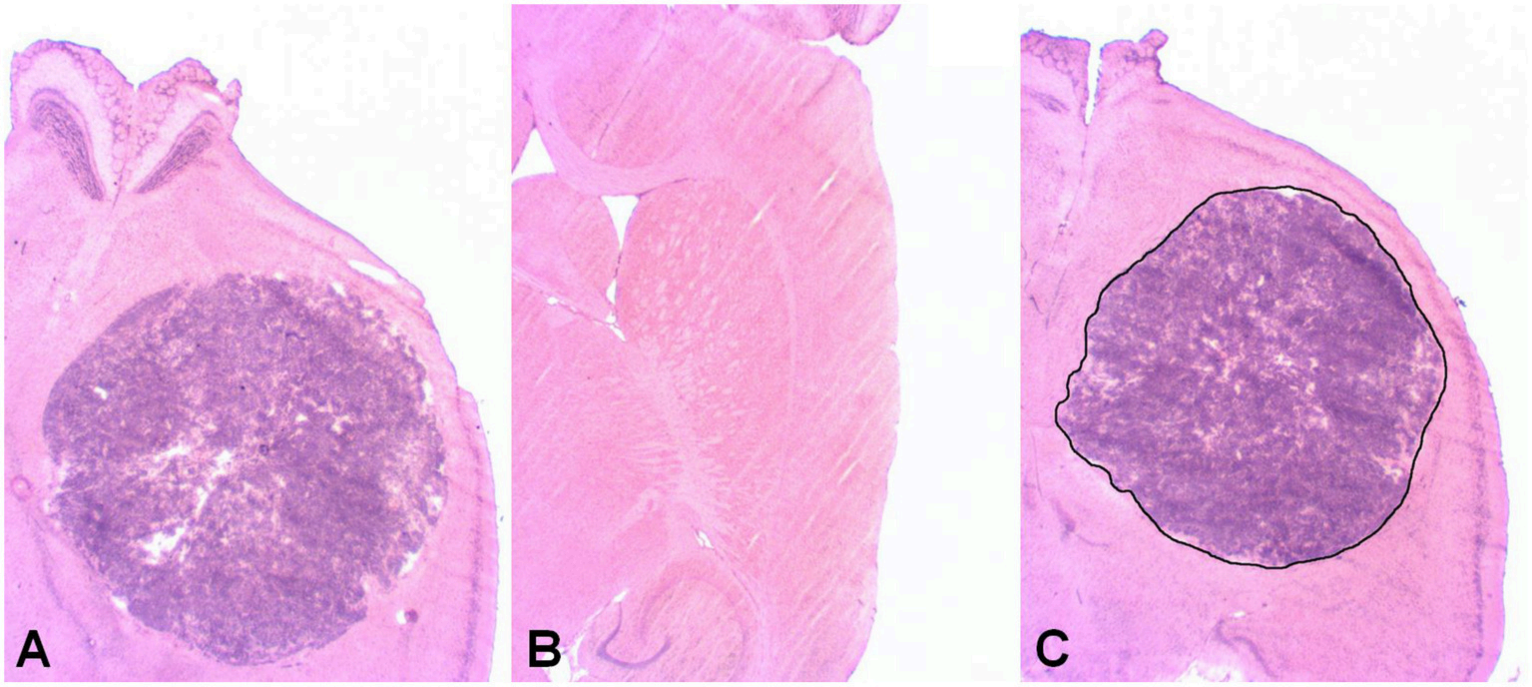
**Hematoxylin and eosin (HE) stain. (A)** Example of a mouse bearing a glioblastoma (GBM) at week-3 post injection. **(B)** Example of a sham operated mouse at the same week-3. The injection site in the right hemisphere shows no histologic evidence for GBM growth or other tissue alterations. **(C)** Example of a manually drawn region of interest (ROI) used for volume calculation according to the Cavalieri method.

All sections were subsequently processed by Hematoxylin and Eosin (HE) staining for histological analyses, either directly after autoradiography or after being temporary stored at −80°C. Overview photographs (see Figure 2) of the tumors were taken with a 3-CCD color video camera (Sony, Tokyo, Japan) attached to an Axioskop 2 microscope (Zeiss, Jena, Germany) with an 1.25x (0.04NA) PlanApo N objective lens (Olympus, Japan). ImageJ (National Institutes of Health, USA) was used to draw the ROIs and to determine their area (see Figure 2C). Histologic tumor volumes were estimated according to the Cavalieri method on the basis of manually drawn tumor ROIs on every 24th section.

### PVEC

PVEC was performed as described previously (Brendel et al., 2014) and adjusted for the GBM mouse model. PVEC was applied to eight GBM mice with confirmed tumor uptake in autoradiography. In particular, we used a VOI-based approach implemented in PMOD (Rousset et al., 1998, 2008) with a full-width-at-half-maximum (FWHM) of 1.20 × 1.20 × 1.72 mm, as evaluated by a stab phantom for the current experimental setup. A VOI-mask containing eight volumes (Figure 1B) was designed for each mouse and consisted of the “optimal” individual tumor VOI (resulting from BTV analyses) and a VOI comprising the rest of the cerebrum, along with an additional six extra-cerebral VOIs (Harderian glands, frontal, superior, basal, spinal, and background). Thus, the main difference of our adjusted approach as compared to application of PVEC in the Alzheimer's Disease model (Brendel et al., 2014) consists in the use of different VOI masks for each individual GBM mouse instead of a single standardized mask for the Alzheimer's Disease mice. Regional point spread functions were calculated through integration of single tissue domains' point spread function and used for computation of weighting factors representing the contributions of the set of eight tissue domains. Coefficients of a geometric transfer matrix (GTM) were calculated, and PVE-corrected radioactivity concentrations were calculated in the defined VOIs by multiplication of the original PET data by the inverted GTM. The PVE-corrected images thus contained only those regions with VOI-mask definitions for comparison with VOI-based results for uncorrected PET data. Finally, VOIs were employed for calculation of PVE-corrected SUVmean/BG.

### BTV

For BTV estimation, a threshold-based VOI assignment was performed. By a range of several thresholds (SUV/BG: ≥1.4; ≥1.6; ≥1.8; ≥2.0) voxels exceeding the respective threshold were included in individual tumor VOIs. For each tumor four different threshold-based PET volumes derived from this calculation and were compared to the histologically determined tumor volume. Additionally, the “optimal” threshold leading to the respective histological tumor volume was determined. To this end, a threshold approximation by using hot 3D VOI assignment was applied until the most appropriate volume was reached. The threshold with highest congruence between PET and histology volumes was defined as “optimal” individual threshold. The resulting VOI was defined as the “optimal” individual tumor VOI (and implemented in the VOI-mask for PVEC as described above). The shape of this “optimal” individual tumor VOI was visually compared to the tumor shape in histology to preclude simple conformity of numerical volume values devoid of geometrical congruence.

### Statistics

Age-related group comparisons of PET results measured as SUVmax/BG, SUVmean/BG and BTV were performed using one-way ANOVA and the Tukey *post-hoc* test for multiple comparisons, calculated by IBM SPSS 22 Statistics. For correlation analyses, Pearson's coefficients of correlation (ρ) were calculated. The association between time and BTV progression was characterized by applying linear, logarithmic, and quadratic regression analyses as implemented in SPSS. The best curve fitting model was determined by the highest ρ-value. A threshold of *p* < 0.05 was considered to be significant.

## Results

### Animal model, study design, and visual assessment

In the course of the study, a total of 43 PET scans was carried out (*n* = 17 in week 2; *n* = 12 in week 3; *n* = 7 in week 4; *n* = 7 in week 5; Table 1). All mice, except two, presented PET-positive tumors at week-3 (9/9), week-4 (5/5), and week-5 (5/5), whereas at week-2 only 25% (3/12) of the animals already had a PET-positive GBM. The two mice where the tumor cells did not grow were excluded from further statistical analysis.

**Figure 3:**
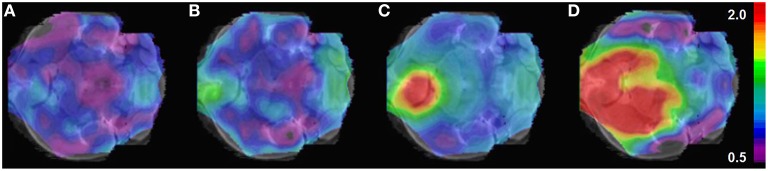
**Example of longitudinal [^18^F]-FET μPET for *in vivo* monitoring of glioma growth. (A)** Week-2 **(B)** week-3 **(C)** week-4 **(D)** week-5 post operationem. SUV/BG (scale bar) is projected on horizontal slices of the mouse brain atlas.

All histologically confirmed tumors could be visually located and clearly demarcated from healthy brain tissue, just as evolution in size and signal strength could be visually tracked over time with PET (Figure 3). No significant PET signal was observed in sham mice when visually comparing the injected side against contralateral. A PET-corresponding autoradiography and histology could be generated for *n* = 2 GBM mice and *n* = 1 sham in week-3, for *n* = 4 GBM mice and *n* = 1 sham in week-5 as well as for *n* = 3 GBM mice and *n* = 1 sham in both week-2 and week-4.

### PET (universal tumor VOI)

**Figure 4:**
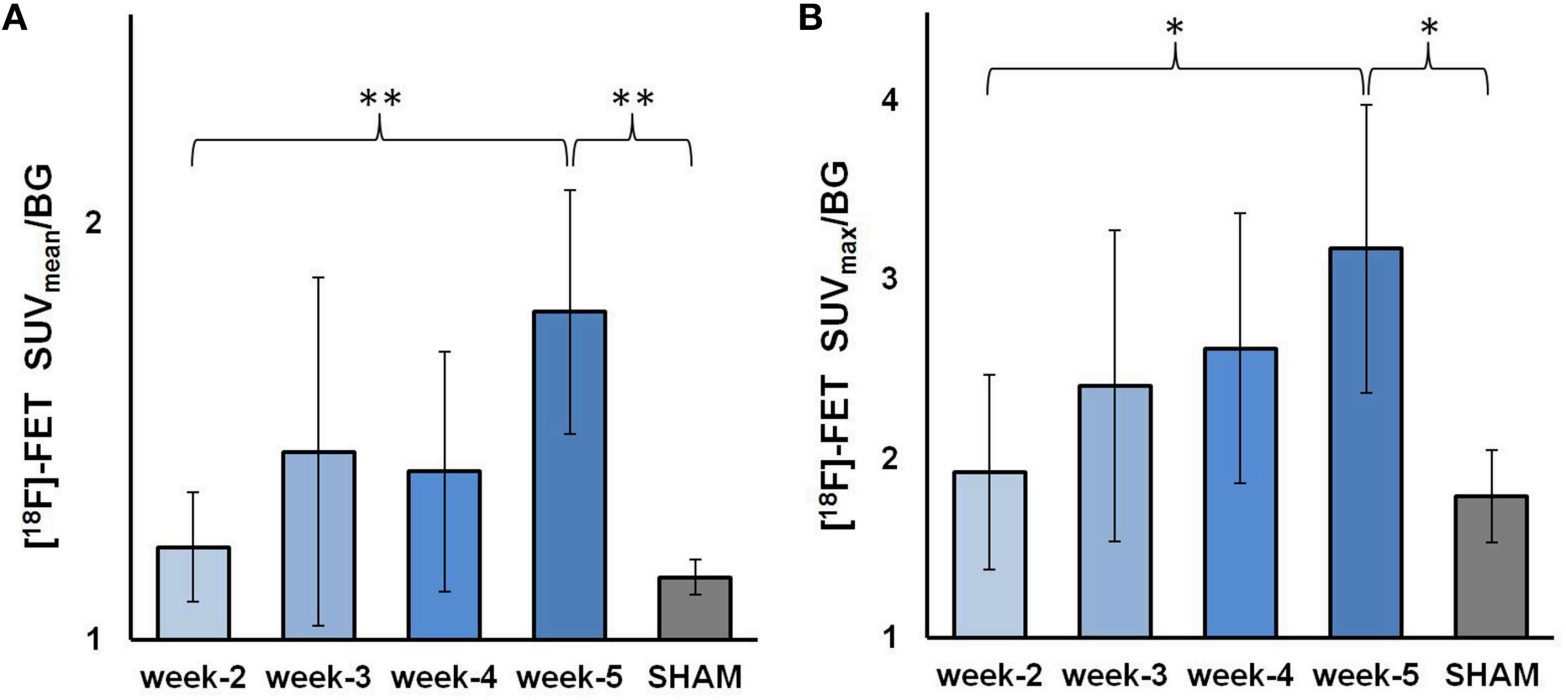
**[^18^F]-FET uptake as assessed by PET using the universal tumor VOI**. The GBM-bearing mice are split in four groups depending on time; the shams are summed up into one group. **(A)** mean SUVmean/BG ± SD **(B)** mean SUVmax/BG ± SD. ^*^p < 0.05; ^**^p < 0.01.

Using the universal tumor VOI for assessment of tracer uptake, the mean SUVmean/BG for GBM mice was 1.22 ± 0.14 at week-2, increased to 1.45 ± 0.41 (*p* = n. s.) at week-3, and 1.40 ± 0.28 (*p* = n. s.) at week-4, finally mounting to 1.78 ± 0.29 (*p* < 0.005) at week-5. Compared to the group of shams (1.15 ± 0.04), the increase of mean SUVmean/BG was +6% at week 2, +26% at week 3, +22% at week 4, and +55% at week 5 (Figure 4A). SUVmax/BG in the GBM mice continually increased over time starting with a mean of 1.92 ± 0.57 at week-2, reaching 2.11 ± 0.87 (*p* = n. s.) at week-3, 2.62 ± 0.76 (*p* = n. s.) at week-4, and 3.18 ± 0.80 (*p* < 0.05) at week-5. In relation to the SUVmax/BG of 1.79 ± 0.26 in the group of shams, the increase was +8% (week-2), +34% (week-3), +46% (week-4), and +77% (week-5; Figure 4B).

In 3/43 scans a relevant spill-in from bone (all frontobasal) influenced SUVmax/BG in the universal target VOI, such that a manual masking was necessary. SUVmax/BG of the tumor was clearly discriminable from spill-in for all those cases, as the spill-in was unexceptionally located at the very edge of the VOI.

### PVEC

**Figure 5:**
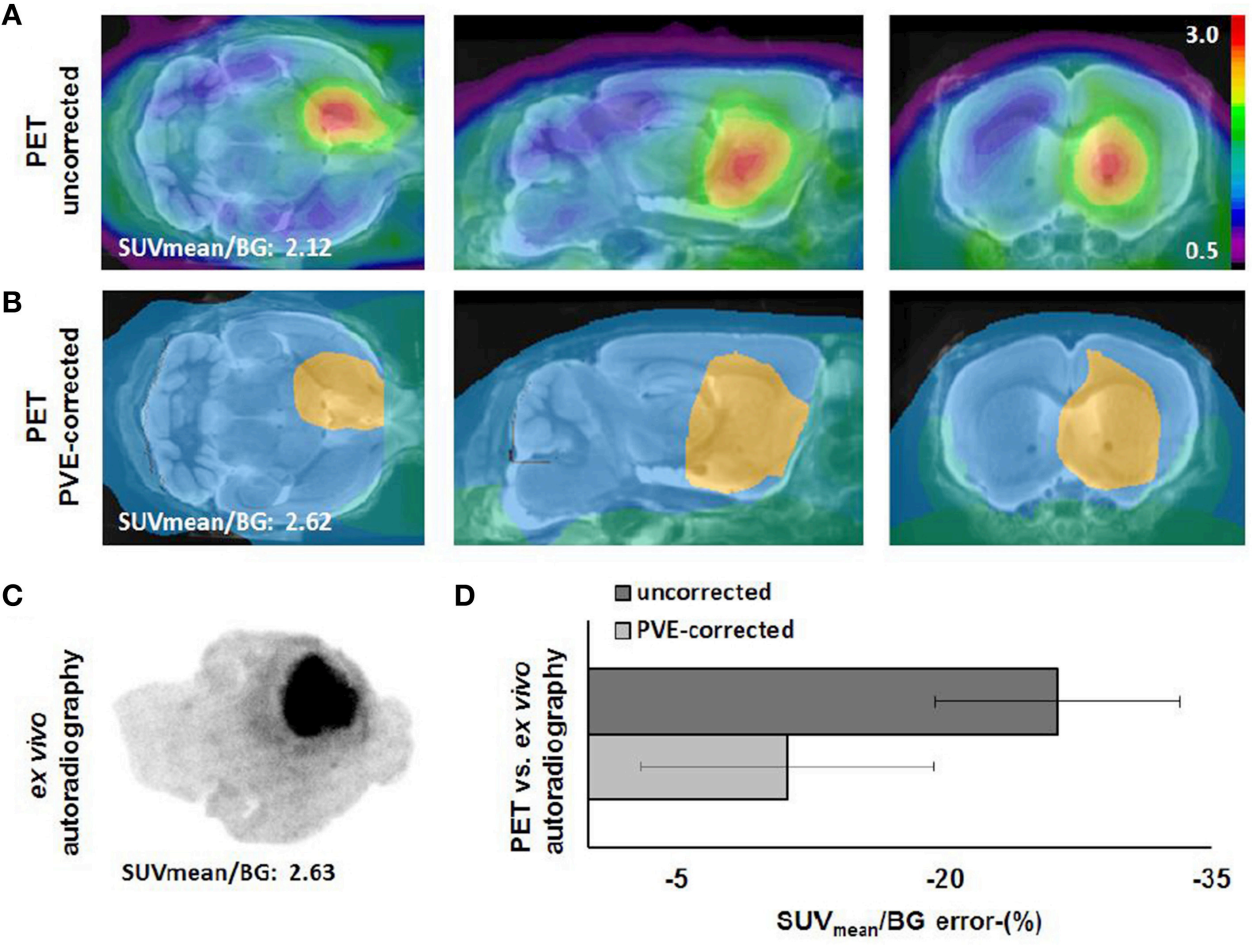
**Comparison of uncorrected [^18^F]-FET PET (upper row, A), PVE-corrected PET (lower row, B) and corresponding *ex vivo* autoradiography (C) of a representative GBM mouse at week-5 post operationem**. PET images were scaled to contralateral tumor-free background and overlain on a 3T MRI mouse template. PVEC was performed with an eight region mask. Error-(%) ± SD of uncorrected (dark bar) and PVE-corrected (light bar) data vs. *ex vivo* autoradiography are shown for the whole group of GBM mice **(D)**.

Compared to the “gold standard” of *ex vivo* autoradiography, SUVmean/BG was assessed by uncorrected PET with an error of −26%. Meanwhile, the error of PET as compared to *ex vivo* autoradiography could be diminished to less than the half (−11%) by applying PVEC as previously described. Figure 5 gives a visual impression of PVEC by opposing both *ex vivo* autoradiography and a PVE-corrected PET image to an uncorrected PET image of a representative GBM mouse at week-5 post operationem.

### BTV estimation

#### Threshold analysis for BTV definition

**Figure 6:**
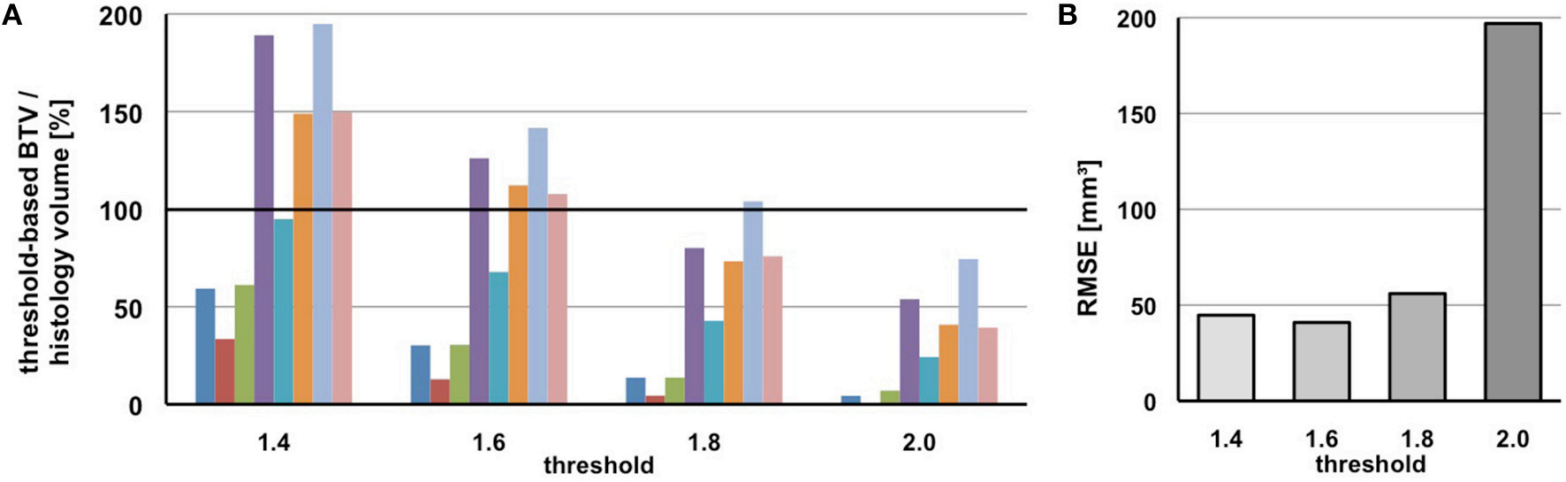
**Biological tumor volume (BTV) as estimated by predefined thresholds deviates from the histology reference volume**. **(A)** Percentage relation of BTV and histology volume is shown for eight mice individually. **(B)** The root-mean-square error for the entire group of mice is shown for each pre-fixed threshold separately.

Figure 6A shows the results of BTV estimation by semi-automatic (predefined) threshold-based VOI calculation in eight mice with PET-positive tumor and comparison of BTV to their individual histology volume serving as reference. Except two estimations with an acceptable error < 5%, all estimated BTVs clearly didn't match with the histology reference. Using 1.4 as threshold factor, only one tumor volume has been estimated with an acceptable error (−5%), while three tumor volumes have been clearly underestimated and four volumes clearly overestimated, two of them considerably (+89, +95%). Using 1.6 as threshold factor, one volume has been overestimated with +8% and three have been clearly overestimated, whereas two volumes have been clearly underestimated, in particular one of them (−87%). Using 1.8 as threshold factor, all volumes—except one accurate estimation with +4% of error—have been clearly underestimated, three of them very highly (−86, −96, −86%). Finally using 2.0 as threshold factor, all the volumes have been clearly underestimated with none of them providing an acceptable estimation of the histology volume.

Therefore, none of these pre-fixed threshold factors qualified to serve as unique standardized threshold for BTV estimation (indicated by high root-mean-square error up to 200%, Figure 6B), even if one of the thresholds might be suitable for BTV estimation in some individual case.

Subsequently we investigated *individual* thresholds for BTV definition using the histology volume as reference, which showed a high variability between the animals (range 1.27–1.83). Hence, no universal threshold for “optimal” volume estimation could be identified.

**Figure 7:**
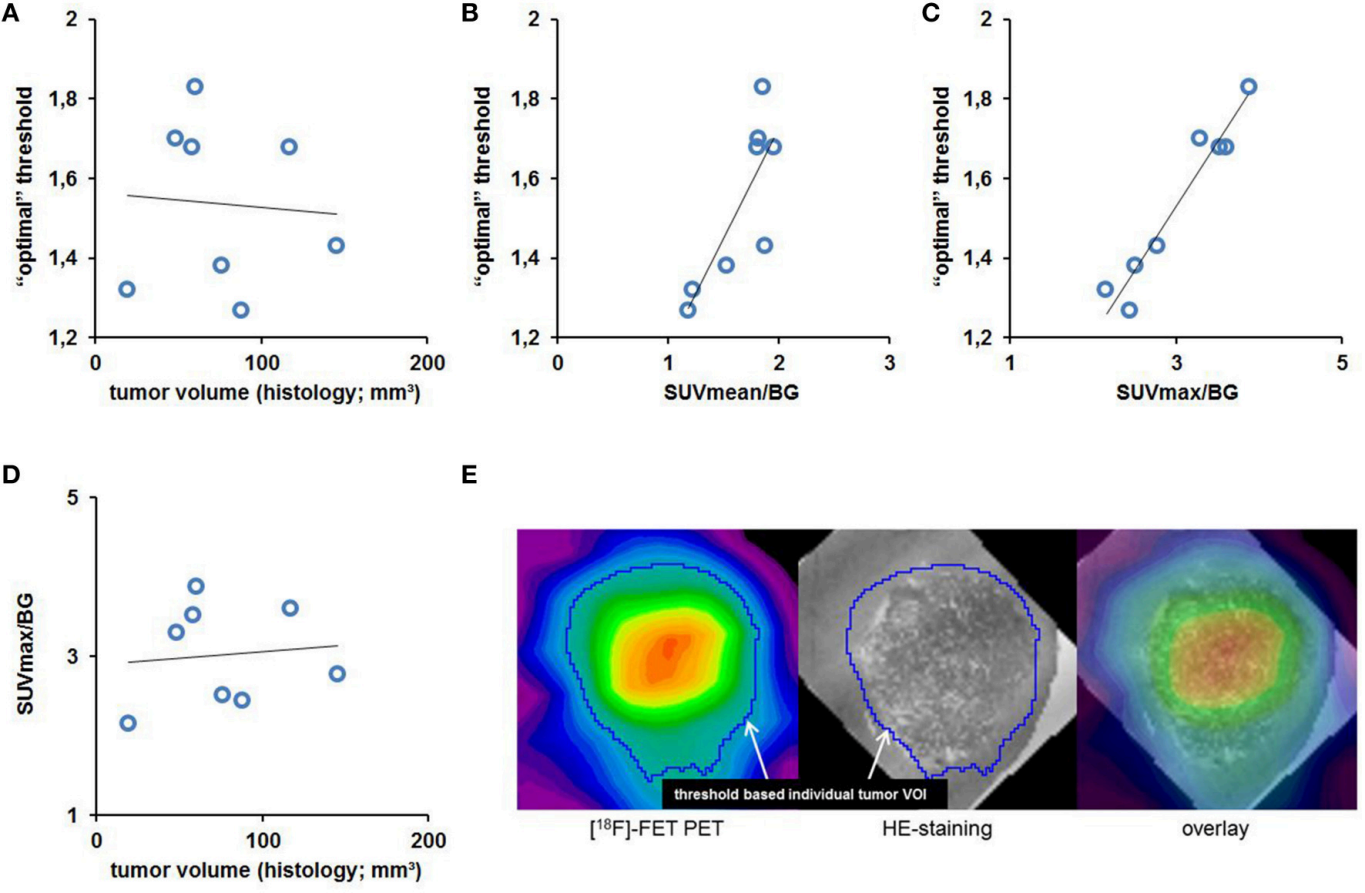
**The individual “optimal” threshold does not correlate with the tumor volume as assessed by histology (A, ρ = −0.07, *p* = n. s.), but shows a strong positive correlation with SUVmean/BG (B, ρ = 0.81; *p* < 0.05) and especially with SUVmax/BG (C, ρ = 0.97, *p* < 0.001)**. The latter however doesn't seem to be dependent on the tumor volume as assessed by histology (**D**, ρ = 0.11; *p* = n. s.). The high congruency between H&E tumor volume and in BTV as defined by the individual “optimal” threshold is illustrated by an image overlay of PET and histology for the tumor of a representative GBM mouse **(E)**.

The “optimal” individual threshold did not correlate with the histology volume (ρ = −0.07, *p* = n. s., Figure 7A), but showed a rather strong positive correlation with SUVmean/BG (ρ = 0.81; *p* < 0.05, Figure 7B) and ultimately revealed a nearly perfect positive correlation with SUVmax/BG (ρ = 0.97, *p* < 0.001, Figure 7C). Being aware of PVE we excluded a strong dependency of PET estimates from the histology-based volume which gave no significant association for SUVmean/BG (ρ = 0.43; *p* = n. s.) and especially SUVmax/BG (ρ = 0.11; *p* = n. s., Figure 7D). The determined VOI shape in PET closely matched to the boundaries obtained from histology (Figure 7E).

The linear association between SUVmax/BG (*x*) derived from uptake analysis in the universal tumor VOI and the “optimal“ individual threshold (*y*) can be expressed by Equation (1) and was consecutively used to obtain BTV in all mice with a detected tumor in PET.

(1) y=0.3215x+0.5654

#### Longitudinal BTV

**Figure 8:**
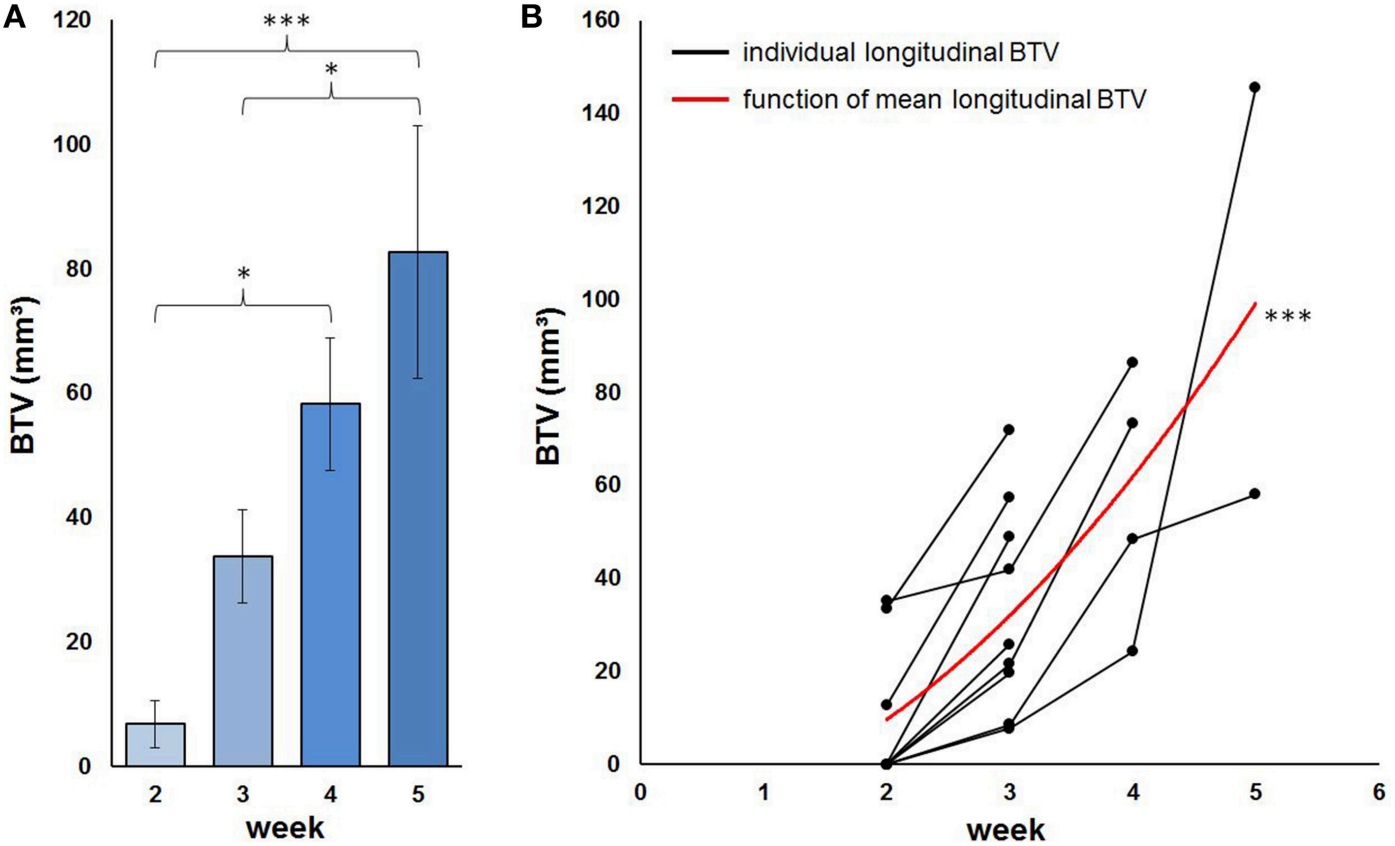
**Progression of BTV in PET as defined by the individual threshold shown as mean BTV per week (A) and furthermore illustrated for each mouse individually together with the mean longitudinal BTV in terms of a quadratic fit (B)**. ^*^p < 0.05; ^***^p < 0.001.

The individual threshold-based method of BTV assessment was applied to the whole dataset and tested with regard to plausibility of longitudinal results and visual control of the resulting individual tumor VOIs (Figure 7E). BTV showed increasing tumor volume with time, starting at week-2 (mean 6.7 mm^3^; range 0–35.1 mm^3^), increasing to a mean BTV of 33.7 mm^3^ at week-3 (range 7.7–57.5 mm^3^, *p* = 0.12), further developing to a mean BTV of 58.3 mm^3^ at week-4 (range 24.2–86.3 mm^3^, *p* < 0.005) and finally terminating at a mean BTV of 82.6 mm^3^ at week-5 (range 38.2–145.7 mm^3^, *p* < 0.001; Figure 8A). The mean increase for all longitudinally assessed volumes was 33.7 mm^3^ per week, however best described by a quadratic fit (ρ = 0.77; *p* < 0.001) that indicated faster progression of BTV at the later stages (Figure 8B).

After application of individual tumor VOIs for the assessment of tracer uptake, 13% (±12%) higher SUVmean/BG values were obtained for the GBM mice with a PET-positive tumor: Mean SUVmean/BG for GBM mice resulted at 1.28 ± 0.32 at week-2, increased to 1.62 ± 0.52 (*p* = n. s.) at week-3, and 1.66 ± 0.41 (*p* = n. s.) at week-4, finally mounting to 1.96 ± 0.34 (*p* < 0.01) at week-5.

SUVmax/BG remained equal after application of individual tumor VOIs. In all mice the supposed tumor edge in PET was captured adequately by a visual control and gave a reasonable differentiation between tumor and background.

### Validation study

**Figure 9:**
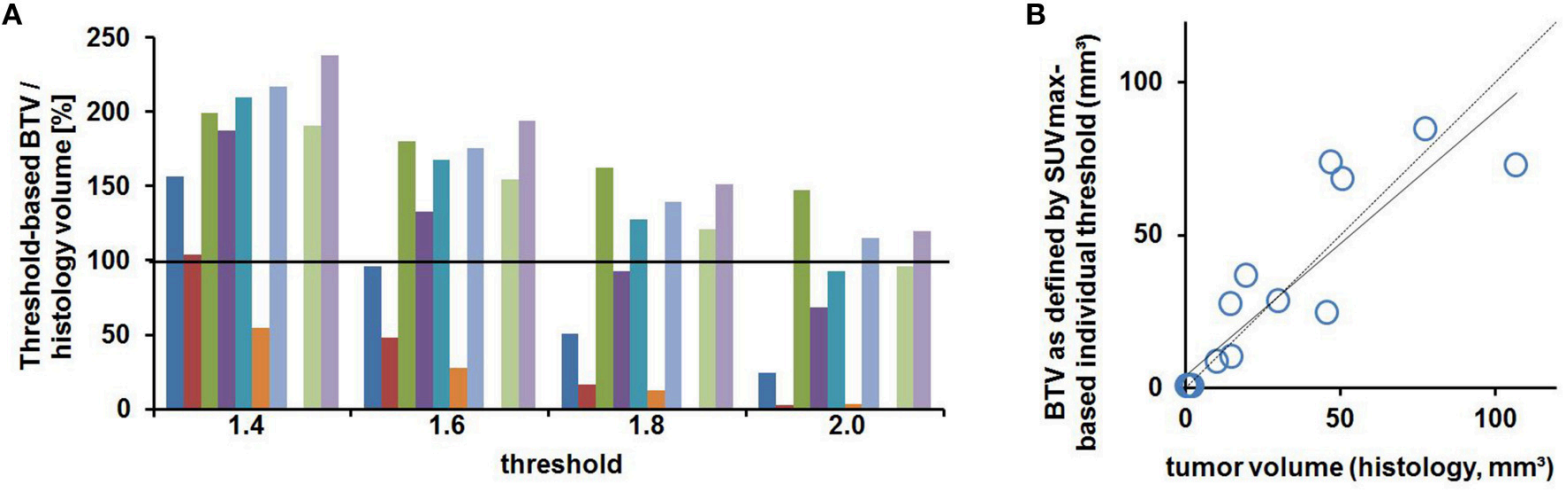
**Validation study. (A)** Percentage relation of BTV and histology volume for predefined thresholds is shown for 10 tumor-bearing mice individually. **(B)** BTV as defined by the new method using SUVmax/BG-based individual thresholds shows a strong correlation with the histology reference volume (*n* = 15, ρ = 0.88, *p* < 0.01).

In accordance with the first experimental round, nearly all mice of the validation round presented PET-positive tumors at week-3 (2/3), week-4 (4/4), and week-5 (4/4), but not at week-2 (0/4). The results of BTV estimation with standardized thresholds (SUV/BG: ≥1.4; ≥1.6; ≥1.8; ≥2.0; *n* = 15) were consistent with the results of the primary study and delivered strongly unreliable BTVs in comparison to the histological reference volumes (Figure 9A).

Finally, Equation (1) (see Section Threshold Analysis for BTV Definition) was applied to all *n* = 15 mice of the validation cohort in order to verify the new SUVmax/BG-based method for individual BTV estimation described above. The resulting individual thresholds led to some highly accurate BTVs (*n* = 9∕15 mice had a deviation of < 7 mm^3^ compared to histology volume) and the overall correlation of BTV estimates to the histology reference volumes was strong in the validation cohort (ρ = 0.88, *p* < 0.01, Figure 9B). Some higher deviations in BTV estimation were observed in larger tumors.

## Discussion

In the present multimodal study, we tested different approaches to perform *in vivo* imaging with small animal [^18^F]-FET PET in an orthotopic glioblastoma mouse model. In addition to assessment of SUVmax/BG and SUVmean/BG, which show a high inter-individual variability and do not correlate with the actual tumor size, we used for the first time threshold-based approaches including several thresholds to determine the BTV in the GBM mouse model. As a major finding, we revealed the common clinical approach of BTV estimation (using standardized thresholds) to be unreliable for volumetric *in vivo* monitoring of tumor growth with [^18^F]-FET PET—at least in this GBM mouse model. In addition, we propose a histology-verified method using *individual* thresholds which improved BTV estimation in comparison to the standard method.

### Animal model and study design

With the initial objective to improve imaging techniques potentially able to survey the response of experimental glioma therapies, we chose the isogenic GL261 cell line (transplanted into C57BL/6 mice) serving as an orthotopic murine brain tumor model, since GL261 is most widely used for the investigation of various antitumoral therapies including gene therapy, immunotherapy or antiangiogenic therapy (Szatmari et al., 2006; Stock et al., 2012).

We included sham-controls for each discrete time point in the study design and observed no [^18^F]-FET PET signal in the tumor-free brain.

Based on the short time between acquisition of PET scans and subsequent brain fixation by intracardiac perfusion, we supposed a high correspondency between PET, histology, and *ex vivo* autoradiography and assumed their direct comparison to be highly convenient. It has to be noted, that the histologic volume assessment has itself some limitations too—slices can for example shrink during the tissue preparation.

### Feasibility, time window, and co-registration

Our data approved general feasibility of [^18^F]-FET imaging using PET in a GBM mouse model. The scanning procedure in its longitudinal setup was well-tolerated by the glioma-bearing mice and provided a copious supply of exploitable PET data.

With a view to our initial targets, e.g. BTV estimation, we opted to concentrate in this study on *static* PET analyses. Pre-clinical glioma research is short on experience regarding adequate time frame setting in [^18^F]-FET PET. Hence, we decided to adopt the time frame from the clinical protocol in glioma patients (20–40 min p. i.; Popperl et al., 2006) and approved its translation to our mouse model—as well as the accurate amount of [^18^F]-FET to inject in mice—by previous pilot scans.

An automatic co-registration would facilitate the most favorable approach for spatial normalization as it as well ensures user independence. Indeed we tried an automatized brain normalization method in PMOD with established parameter of our group (Overhoff et al., 2016). However, as [^18^F]-PET has a relatively low uptake in the not-tumor-affected brain we observed a significant influence of the higher and variable extracerebral uptake on results of the automatic approach. Up to now the results were not satisfactory enough to introduce this method to the work-flow and we circumvented this issue by blinding the mice to the reader and used a fine realignment of individual mice to their baseline. Visual inspection of the resulting manually co-registered images gave good fits to the brain borders of the MRI mouse atlas and should serve as a precise method.

### Visual examination

According to our results, *in vivo* monitoring of the inoculated tumor is feasible with longitudinal [^18^F]-FET PET by simple means of visual examination as a first simplistic and reader-dependent approach (Figure 3). PET-positive tumors were found in 25% of the GBM mice examined in the second week; negative reads were confirmed by negative histology (3/3 negative). The smallest histologically verified tumor (19 mm^3^, week-3) was easily identifiable by a visual read such as the smallest assessed tumor by the BTV threshold method (7 mm^3^, week-3). These results are consistent with [^18^F]-FET being a highly sensitive biomarker for glioma tissue leading to a sharp contrast between tumor and healthy background. However, as a matter of course, quantification is mandatory to objectify previous findings and enable a reader-independent consideration of tumoral lesions and growth monitoring with [^18^F]-FET PET. This turns out to be especially important, when even slight differences in uptake behavior or tumor size—e.g. in the context of therapy studies—have to be distinguished.

### Definition and limitations of standardized VOIs

According to the common approach, we first determined SUVmax and SUVmean in a predefined uniform target VOI surrounding the tumor and corrected both values for healthy background (i.e., SUVmean of the tumor-free VOI in the contralateral hemisphere). At this point, the dimensions of our chosen tumor-free background VOI (123 mm^3^, Figure 1A) deserve particular notice, since it overpasses other reported background VOIs 30 times in size and therefore guarantees a reliable background quantification (Nedergaard et al., 2014, 2015). Due to this extent, activity fluctuations in healthy brain tissue as well as single accidental voxels of bone spill-in (since [^18^F] itself is known to accumulate in the cranium to a certain extent; Mille et al., 2012) shouldn't have remarkable consequences on the background's SUVmean.

The above-sketched approach (using standardized target VOIs) is reasonable for assessment of SUVmax/BG since the universal tumor VOI should compromise the entire tumor and consequently the voxel with maximum [^18^F]-FET uptake. Care has to be taken when mice exhibit such relevant bone uptake that the universal target VOI gets contaminated, which was the case in 3/43 scans. Since a single contaminated voxel determines an overestimated SUVmax/BG we recommend manual control slice-by-slice, which is easily feasible by a screening ROI/VOI for the maximum uptake. This is essential when SUVmax/BG is considered for the definition of individual thresholds for BTV assessment. However, in all three contaminated cases the maximum related to the tumor was clearly distinguishable from the maximum spill-in from bone and therefore we do not see a major limitation caused by this issue. The assessment of SUVmean/BG implicates limitations since a universal tumor VOI doesn't account for the different tumor sizes, but the related SUVmean/BG however accounts for every compromised voxel in equal measure. Thus, SUVmean/BG will be underestimated in small tumors due to a relatively high portion of healthy brain tissue comprised by the large universal VOI. Our universal target VOI after all embraces advanced gliomas but therefore doesn't come up in an optimum way to the smaller ones.

### Uptake analyses

The standard PET parameters show a clear temporal progress in gliomas in this mouse model (Figure 4). We interpret this finding as naturally related to tumor development and as being on this note typical for the evolution of tumoral [^18^F]-FET uptake with time as well in human data. However, it should be noticed well that the pathophysiological contributions leading to an elevated peak (SUVmax/BG) or global (SUVmean/BG) tumor uptake of [^18^F]-FET are not fully understood and are probably caused multifactorial by the molecular features of the tumor cells. Indeed we find an inter-individually heterogeneous [^18^F]-FET uptake in this cohort of mice, with some animals bearing high uptake tumors even at week-2 post operationem and others that indicate a slow development of low uptake tumors over the whole longitudinal setup. The heterogeneity is further boosted by independency of [^18^F]-FET uptake from tumor sizes (Figure 7D), which implicates that large tumors do not necessarily need to be characterized by a high tumor uptake and vice versa. This finding shows that measurement of SUV might reflect molecular features of the tumor but not tumor growth in terms of size, which would be of interest when evaluating treatment responses. The reason for this inter-individual uptake variability of SUVmax/BG and SUVmean/BG remains to be investigated, but surely the heterogeneity represents a chance for better understanding the functionality of [^18^F]-FET uptake by intensified basic research on [^18^F]-FET PET in GBM mouse models.

In this context, the methodology itself should as well be considered as a contributor to the heterogeneity as the above-discussed insufficient fitting accuracy of the chosen universal target VOI could potentially induce under- and overestimations of [^18^F]-FET uptake values. Nonetheless even with application of *individual* tumor VOIs to all mice (as discussed in more detail below) the variance of SUVmean/BG did not decrease and indicated that the heterogeneity is rather a natural feature of the model but not a methodological bias. This and the finding, that the SUV parameters did not correlate with the actual tumor volume, emphasizes even more that stand alone SUVmax/BG or SUVmean/BG assessments are not ideal for *in vivo* characterization of glioblastoma as they do not reflect all important *in vivo* information of the tumor.

### PVEC

By applying PVEC to our PET data, we succeeded to improve accuracy of [^18^F]-FET PET by shortening the gap between PET and autoradiography findings which are caused by the limited resolution of the PET scanner (Visser et al., 2009). Despite decreasing errors of SUVmean/BG by this method, it has to be considered that only a tumoral and a background SUV/BG result after PVEC, which limits regional analyses.

### BTV

As a major finding of this study, we revealed the common clinical approach of semi-automatic threshold-based BTV estimation by [^18^F]-FET PET to deliver apparently inconsistent results when correlated with the gold standard histology volume—at least in the present GBM mouse model (Figure 6). It was by intention, that we included the thresholds previously used in the clinical field for BTV estimation (Jansen et al., 2012b, 2013; Suchorska et al., 2015) in our analyses, however none of those pre-fixed thresholds was suitable to mirror PET-based BTVs consistently with the tumor volume as assessed by histology. As human studies hardly have the possibility to assess the “real” BTV by gold standard histology assessment directly after imaging, we want to emphasize the value of pre-clinical studies regarding such issues. Human studies have compared PET-derived BTV to tumor volumes on MRI and have reported substantial discrepancies between both imaging methods (Ewelt et al., 2011; Niyazi et al., 2011; Suchorska et al., 2015). A limitation of our study is the lacking μMRI, which would have facilitated a direct comparison of functional and structural imaging methods for BTV estimation. Nonetheless, there are only few facilities that have the opportunity of pre-clinical hybrid imaging (serial or simultaneously) so far, clearly justifying current stand-alone μPET investigations. Furthermore, numerous studies have revealed that the delineation of vital tumor tissue is better with [^18^F]-FET PET than MRI in glioma patients, particularly after multimodal treatment involving treatment-induced changes on MRI which cannot be accurately differentiated from tumor tissue (Galldiks et al., 2012b, 2013a,b, 2015a; Jansen et al., 2013).

Since the common approach for BTV estimation with [^18^F]-FET PET after all did not seem to be appropriate for the GBM mouse model, we determined the “optimal” *individual* threshold for BTV definition using the histology volume as reference for each mouse. These thresholds showed a high variability, but surprisingly, SUVmax/BG correlated nearly perfectly with these individual thresholds (Figure 7C). We excluded partial volume effects as the key actor of the correlation as neither SUVmax/BG nor the individual threshold seem to depend on the histology tumor volume (Figures 7A,D). Thus, the SUVmax/BG might adequately resemble the magnitude of [^18^F]-FET uptake in the majority of tumor cells in the individual mouse, including those cells at the tumor's edge, and thereby determine the threshold between tumor and healthy brain tissue. We conducted a subsequent validation study with twice the number of mice (*n* = 15) to test the reproducibility of our novel approach. Altogether, the method led to accurate BTV estimations in both rounds (total *n* = 23), which, again, was clearly superior to the BTV estimation using predefined thresholds. Some outliers in large scaled tumors might limit the method with regard to accuracy in very large volumes. Nonetheless, the method strongly improved BTV estimation when compared to the standard approach (using standardized thresholds) and mainly delivered accurate approximations of histology volume for this stand-alone μPET approach.

Besides various thresholding-approaches, shape-based approaches as well have been investigated for BTV definition in rodent tumor models: Wu et al. described—amongst three different threshold-based approaches—an edge-detection-based automated contouring system for definition of BTV using [^18^F]-FDG PET in mammary tumors (grown in Lewis rats). In contrary to their threshold-based approaches, the shape-based system failed to produce reliable volumes in comparison to the histology volume due to the system's lack of reproducibility (Wu et al., 2015). However, we share the author's opinion on the relevance of edge detection methods: In this study, we do not provide a shape-based method, but—on this note—controlled the shape of every PET-delineated volume resulting from our individual method with the tumor shape in histology and revealed both shapes to closely match.

Although [^18^F]-FET is the most established tracer for molecular glioma imaging in the clinical field, other tracers have shown promising results for delineation of BTV in the pre-clinical field as well: ^11^C-methylaminoisobutyric acid ([^11^C]-MeAIB) has for example been used in a double tracer study together with [^18^F]-FDG to estimate tumor volumes in two phenotypically different orthotopic GBM rat models. [^11^C]-MeAIB PET was accurate for volume estimation in non-infiltrating brain tumors when compared to histologic findings, proved however to be hardly reproducible in highly infiltrating brain tumors (Halle et al., 2015).

As in our study, the authors primarily focused on thresholding methods, which are the most commonly used methods for image segmentation in regard to volume estimation. It would be of interest for future studies, to investigate how less commonly used segmentation methods, using e. g. edge detection, region growing, clustering, stochastic models, deformable models, or classifiers (Zaidi and El Naqa, 2010), could be used to improve the results of our new approach.

## Conclusions

[^18^F]-FET PET is feasible for glioma imaging in the GBM mouse model. Standard PET parameters reflect tumor growth intra-individually, but GL261 tumor cells show high inter-individual uptake variability and standard uptake values do not correlate with tumor size. PVEC is beneficial to improve accuracy of [^18^F]-FET PET by reducing the error of SUVmean/BG as assessed by PET in comparison to *ex vivo* autoradiography. Standardized thresholds respective to the common approach seem to be inappropriate for BTV estimation in gliomas—at least in the present glioblastoma mouse model. Individualized thresholds on the contrary seem to be more appropriate for volumetric *in vivo* monitoring of tumor growth with [^18^F]-FET PET.

## Author contributions

SG, GM, RK, and RG accounted for establishment of cell culture and animal model. AH, SG, JC, and GM accounted for animal care. AH and SG accounted for cell inoculation and perfusions. AH, SG, and RK accounted for acquisition of histology. AH and MB accounted for acquisition of autoradiography. AH, MB, JC, EM, GB, MU, FJG, AR, PB, REK, RG and NLA accounted for conception and design of the study. MB, EM and GB accounted for PET physics. FJG accounted for radiochemistry. AH, MB, and AR accounted for PVEC. AH, MB, EM, GB, MU, FG, AR, PB, RK, RG, and NA accounted for analysis and interpretation of data.

### Conflict of interest statement

The authors declare that the research was conducted in the absence of any commercial or financial relationships that could be construed as a potential conflict of interest.
